# Development of a Nomogram Model Predicting Current Bone Scan Positivity in Patients Treated with Androgen-Deprivation Therapy for Prostate Cancer

**DOI:** 10.3389/fonc.2014.00296

**Published:** 2014-10-27

**Authors:** Geoffrey T. Gotto, Changhong Yu, Melanie Bernstein, James A. Eastham, Michael W. Kattan

**Affiliations:** ^1^Southern Alberta Institute of Urology, University of Calgary, Calgary, AB, Canada; ^2^Department of Quantitative Health Sciences, Cleveland Clinic Foundation, Cleveland, OH, USA; ^3^Urology Service, Department of Surgery, Memorial Sloan Kettering Cancer Center, New York, NY, USA

**Keywords:** non-steroidal anti-androgens, radionuclide imaging, nomogram, prostatic neoplasms, androgen-deprivation therapy, bone scan positivity

## Abstract

**Purpose**: To develop a nomogram predictive of current bone scan positivity in patients receiving androgen-deprivation therapy (ADT) for advanced prostate cancer; to augment clinical judgment and highlight patients in need of additional imaging investigations.

**Materials and methods**: A retrospective chart review of bone scan records (conventional ^99m^Tc-scintigraphy) of 1,293 patients who received ADT at the Memorial Sloan-Kettering Cancer Center from 2000 to 2011. Multivariable logistic regression analysis was used to identify variables suitable for inclusion in the nomogram. The probability of current bone scan positivity was determined using these variables and the predictive accuracy of the nomogram was quantified by concordance index.

**Results**: In total, 2,681 bone scan records were analyzed and 636 patients had a positive result. Overall, the median pre-scan prostate-specific antigen (PSA) level was 2.4 ng/ml; median PSA doubling time (PSADT) was 5.8 months. At the time of a positive scan, median PSA level was 8.2 ng/ml; 53% of patients had PSA <10 ng/ml; median PSADT was 4.0 months. Five variables were included in the nomogram: number of previous negative bone scans after initiating ADT, PSA level, Gleason grade sum, and history of radical prostatectomy and radiotherapy. A concordance index value of 0.721 was calculated for the nomogram. This was a retrospective study based on limited data in patients treated in a large cancer center who underwent conventional ^99m^Tc bone scans, which themselves have inherent limitations.

**Conclusion**: This is the first nomogram to predict current bone scan positivity in ADT-treated prostate cancer patients, providing high predictive accuracy.

## Introduction

Between 15 and 40% of patients treated for localized prostate cancer will experience biochemical recurrence (BCR), as shown by rising prostate-specific antigen (PSA) levels (1). Androgen-deprivation therapy (ADT) is an accepted standard of care for patients who have received therapy with curative intent and subsequently show systemic relapse (2). Castration-resistant prostate cancer (CRPC) is defined as disease progression, typically identified by rising PSA levels and/or worsening disease according to imaging, despite castration levels of testosterone; most patients with CRPC will eventually develop metastatic (M1) disease (3).

The axial skeleton is the most common site of systemic metastasis in patients with prostate cancer (4). Recent prospective studies demonstrated a median time of approximately 25–30 months from CRPC to M1 CRPC (3, 5, 6). M1 bone disease is associated with significant morbidity, including pain, impaired mobility, and pathological fractures (7). Bone imaging can provide important information on the clinical status of asymptomatic patients with rising PSA levels. In addition, novel therapies, some of which provide benefits for patients with asymptomatic M1 CRPC, have recently been developed and approved (8, 9).

Although higher PSA levels and more rapid PSA doubling times (PSADT) are associated with a shorter time to bone metastasis (5, 10), the National Comprehensive Cancer Network prostate cancer guidelines provide minimal guidance on when to start and how often to repeat bone scan imaging in BCR patients (11). A recent study suggests that many patients classified as having non-metastatic (M0) disease are not undergoing early imaging and detection of the transition to the M1 state. In the ENTHUSE trial, a phase III trial enrolling men with M0 CRPC, 32% of screened men actually had M1 disease (based on magnetic resonance imaging, computed tomography, or bone scan results) (12).

Nomograms can provide individualized, disease-specific risk estimations that aid clinical management decisions (13). However, no nomogram is currently available to predict bone scan positivity in patients receiving ADT, information that could prompt relevant imaging investigations. We therefore investigated disease and treatment-related factors that could be predictive of metastases in this patient population, and used these factors to develop a nomogram model. To achieve this, we reviewed the records of a large database of patients treated at a single institution over an 11-year period who underwent conventional ^99m^Tc-scintigraphy.

## Materials and Methods

This retrospective chart review included patients treated at the Memorial Sloan-Kettering Cancer Center for prostate adenocarcinoma from 2000 to 2011. All patients with bone scan records who had received ADT during this period were included in the study, with the exception of patients participating in clinical trials and those who had received prior chemotherapy or estrogen therapy (which could affect the time to bone scan positivity). Data on the frequencies of visits and reasons for the bone scans being performed were not collected. Patients were followed up from the initiation of ADT until either the first positive bone scan or the last hospital visit. Patients may have received either intermittent or continuous ADT, but these data were not collected. Data on the use of calcium, vitamin D, or bisphosphonates were not collected as these were not expected to affect bone scan positivity.

A chart review of patients’ records was undertaken. A scan was coded “positive” if terms referring to metastases were identified in the records, “negative” if no such terms were found, and “unknown” if records had equivocal findings. Based on clinical relevance and data availability, eight disease and treatment variables were evaluated as potential predictors of a positive ^99m^Tc bone scan: number of previous negative bone scans; current PSA level; PSADT; most recent Gleason grade sum (at most recent biopsy or at prostatectomy); and a history of prior radical prostatectomy, radiotherapy, brachytherapy, or cryotherapy. PSADT was calculated based on all PSA data points measured prior to each bone scan and the interval(s) between them; negative PSADT indicated a decrease in PSA level from the previous reading. The radiotherapy group included all patients who received treatment related to the prostate. Missing values were multiply imputed before conducting statistical analyses. Testosterone data were not available for all patients and were not included in the analysis.

Multivariable logistic regression analysis was used to determine which factors should be included in the nomogram model. Restricted cubic splines were applied to continuous or nominal variables with the purpose of relaxing the commonly assumed linear association between risk factors and the outcome. Variables were selected using the step-down model reduction method (14) and identified predictors were included in the final parsimonious model on which the nomogram was built. Generalized estimating equations were used to handle clustering bone scans from the same patient. The current probability of a positive bone scan was determined by the nomogram using the patient variables at the time of each bone scan, and the predictive accuracy of the model was quantified using Harrell’s concordance index (C-index) (15). The C-index is equivalent to the area under the receiver operating characteristic curve and values can range from 0.5, which indicates no predictive discrimination, to 1.0, which denotes a perfect separation of patients with different outcomes (16, 17). Bootstrapping with 1,000 resamples was utilized to correct over-fitting bias for both the model discrimination and calibration evaluations. All statistical analyses and graphics were conducted using the open-source statistical software R version 2.14.2 (R Foundation for Statistical Computing, Vienna, Austria) in combination with the additional packages Design (Frank Harrell, Vanderbilt University, Nashville, TN, USA) and Zelig (Harvard University, Cambridge, MA, USA). The statistical hypothesis test was considered to be significant if *p* < 0.05.

## Results

A total of 1,293 patients received ADT at the Memorial Sloan-Kettering Cancer Center between 2000 and 2011 and underwent at least one ^99m^Tc bone scan during the median follow-up period of 54 months (range 3.4–144 months) from ADT initiation; 869 patients had at least one scan during ADT. Of the 424 patients who had either no scan during ADT or an unknown ADT end date, 298 had a post-ADT scan with a known ADT end date. Overall, 2,681 bone scan records were analyzed and the disease characteristics at the time of each bone scan are shown in Table 1.

**Table 1:** **Patient and disease characteristics at each bone scan (*n* = 2,681)**.

Median (range) PSA level prior to each bone scan, ng/ml	2.4 (0–4,648)
Median (range) PSADT prior to each bone scan, months^a^	5.8 (−120–120)
Scan result, *n* (%)
Positive	636 (23.7)
Negative	2,045 (76.3)
Most recent Gleason grade sum, *n* (%)
≤6	267 (10.0)
7	944 (35.2)
8	606 (22.6)
9	787 (29.4)
10	63 (2.3)
Missing	14 (0.5)
Pre-scan radiotherapy, *n* (%)^b^
Yes	1,390 (51.8)
No	1,291 (48.2)
Pre-scan radical prostatectomy, *n* (%)
Yes	872 (32.5)
No	1,809 (67.5)
Pre-scan brachytherapy, *n* (%)
Yes	202 (7.5)
No	2,479 (92.5)
Pre-scan cryotherapy, *n* (%)
Yes	25 (0.9)
No	2,656 (99.1)

*^a^PSADT was calculated based on the PSA levels measured prior to each bone scan; negative PSADT values indicated a decrease in PSA levels from the previous reading. Median (range) number of PSA values used was 13 (2–99); 95.3% of PSADT was calculated using 3 or more PSA values*.

*^b^The radiotherapy group included all patients who received treatment related to the prostate*.

Of the 1,293 patients included in the study, 636 had a positive bone scan (Table 2). A median of 0 (range 0–15) negative scans were conducted after the initiation of ADT but prior to obtaining a positive scan, and the median time between the last negative scan and the positive scan was 6.7 months (range 0.5–46.7 months). Median PSA level prior to each positive bone scan was 8.2 ng/ml (range 0–4,648 ng/ml) and median PSADT was 4.0 months (range -120 to 120 months). Around half (53%) of the patients had PSA levels <10 ng/ml at the time of their first positive scan.

**Table 2:** **Patient and disease characteristics at the time of the first positive scan (*n* = 636)**.

Median (range) PSA level prior to each bone scan, ng/ml	8.2 (0–4,648)
PSA level (ng/ml) category, *n* (%)
<5	271 (42.6)
5–10	68 (10.7)
10–20	57 (9.0)
20–50	82 (12.9)
50–100	52 (8.2)
≥100	106 (16.7)
Median (range) PSADT prior to each bone scan, months	4.0 (−120–120)
Median (range) number of prior negative scans after initiating ADT	0 (0–15)
Median (range) time since last negative bone scan, months	6.7 (0.5–46.7)
Pre-scan radiotherapy, *n* (%)
Yes	242 (38.1)
No	394 (61.9)
Pre-scan radical prostatectomy, *n* (%)
Yes	147 (23.1)
No	489 (76.9)

**Figure 1:**
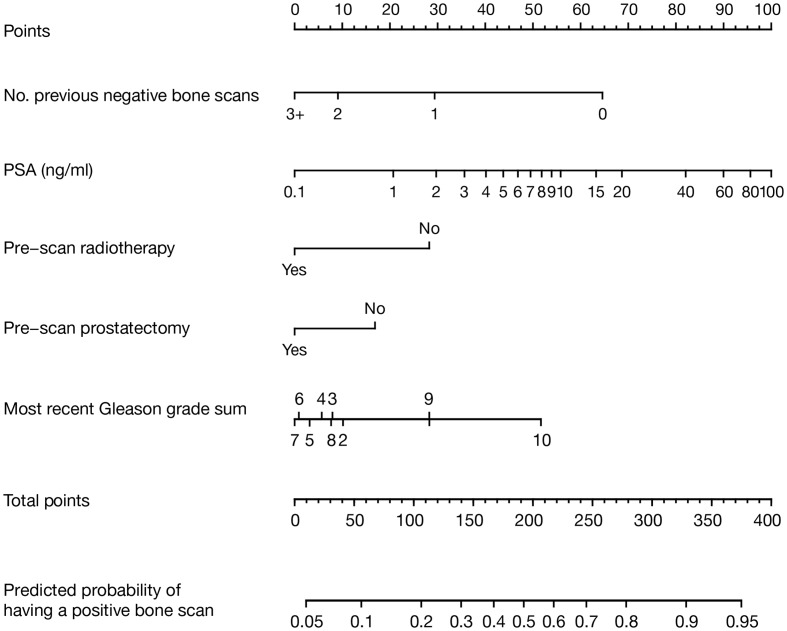
**Nomogram model developed to predict current bone scan positivity in patients treated with ADT for prostate cancer**. The result for each variable has a corresponding points score (top scale). The points score for each variable is determined and summed to calculate the total points for a given patient. This value is located on the total points scale (second from the bottom). The predicted probability of having a current positive bone scan is determined by drawing a vertical line down from the total points scale to the probability scale below. Number of previous negative bone scans should only include those after the initiation of ADT.

Multivariable logistic regression analysis revealed that five variables were significant predictors of having a positive bone scan: low number of previous negative bone scans after initiating ADT, high PSA levels, high Gleason grade sum, and having no history of radical prostatectomy or radiotherapy (Table 3). These variables were then incorporated into the nomogram model. Brachytherapy, cryotherapy, and PSADT were not predictive of a positive scan (data not shown) and were not included. The final nomogram model is shown in Figure 1.

**Table 3:** **Multivariable logistic regression analysis of the variables included in the nomogram model**.

Variable	Comparison	Odds ratio^a^ (95% CI)	*p*-value
PSA level (ng/ml)	13.7 vs. 0.2^a^	2.47 (2.07–2.93)	<0.01
Number of previous negative bone scans after initiating ADT	2 vs. 0^a^	0.41 (0.33–0.51)	<0.01
Most recent Gleason grade sum	9 vs. 7^a^	1.58 (1.29–1.93)	<0.01
Pre-scan radical prostatectomy	Yes vs. No	0.63 (0.52–0.78)	0.01
Pre-scan radiotherapy	Yes vs. No	0.76 (0.60–0.97)	0.03

*^a^For continuous variables, the third quartile (Q3) and first quartile (Q1) are shown and were compared*.

**Figure 2:**
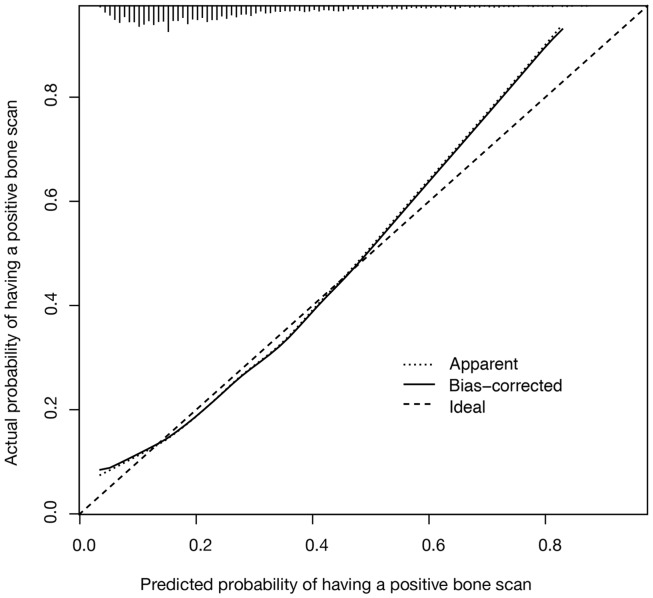
**Calibration plot for the nomogram model showing predicted vs. actual likelihood of having a positive bone scan is shown**. The vertical lines along the top of the figure show the distribution of nomogram-predicted probabilities for patients in the study cohort.

A C-index value of 0.721 was calculated for the nomogram model. As shown in Figure 2, a close relationship was observed between the results achieved with the nomogram model and the actual clinical findings (ideal calibration result).

## Discussion

The nomogram model presented in this study is the first analytical tool developed to predict current bone scan positivity in prostate cancer patients treated with ADT. A C-index value of 0.721 was calculated for the nomogram model, indicating high predictive accuracy. This simple nomogram could therefore be used to highlight patients in the clinic who should be a high priority for bone scan. This could offer substantial benefits to patients, as earlier detection of the transition from M0 to M1 CRPC may allow earlier therapeutic intervention.

Current National Comprehensive Cancer Network guidelines state that a bone scan is appropriate in men with post-prostatectomy BCR (i.e., patients who have not received ADT) when symptoms develop or PSA levels increase rapidly (11). However, to date, there has not been sufficient clinical evidence to provide recommendations on imaging in men treated with ADT and, at present, the transition of patients from M0 to M1 CRPC is frequently missed (12). In the absence of detailed guidance on when to initiate imaging in ADT-treated patients with prostate cancer, our nomogram therefore has important clinical applications. For example, it could reduce potential physician subjectivity, and highlight patients who should receive an additional bone scan. Indeed, there is evidence across various therapeutic areas that nomograms can be an effective tool in predicting clinical outcomes in individual patients (18–21), with one nomogram model predicting future bone scan positivity in prostate cancer patients who had not received ADT more accurately than a group of expert clinicians (C-index values 0.812 vs. 0.628) (22). There is therefore a strong argument to use nomograms to augment clinical judgment and prompt further imaging investigations.

During development of the nomogram, five factors were identified as predictive of current bone scan positivity: lower number of previous negative bone scans after initiating ADT, higher PSA level, higher Gleason grade sum, and having no history of radical prostatectomy or radiotherapy. This is generally in line with previous findings in prostate cancer. Higher PSA levels ( >10 ng/ml) have been associated with a shorter time to first bone metastasis in CRPC (5, 10), as well as decreased overall survival (10). Several studies have also demonstrated the association between higher Gleason scores and an increased risk of bone metastases in patients with newly diagnosed prostate cancer (23–25). Furthermore, Gleason score and current PSA levels were significant predictors of systemic progression in patients who had received previous ADT or radiotherapy (26). Although these factors do yield useful prognostic information, there is a clear need for new predictors to be identified in future studies.

Data suggest that patients who have received ADT may have a different risk of bone metastasis than those who have not, and this should be taken into account when monitoring PSA levels. In our study, PSA level at the time of a positive scan appeared to be highly variable (range 0–4,648 ng/ml); however, the proportion of patients with PSA levels <10 ng/ml at the time of positive bone scan was high at 53%. A similar analysis in men who had not received ADT found that PSA levels were lower at the time of M1 diagnosis (25.9% of patients had PSA <10 ng/ml, 50.8% had PSA 10–100 ng/ml, and 23.3% had PSA >100 ng/ml) (27). In another study, men with BCR on ADT were significantly more likely to have a positive scan than patients who had not received ADT, when the results were adjusted for PSA level, PSADT, and PSA velocity (odds ratio 5.00; *p* = 0.004) (28). These findings suggest that data obtained in patients who have not received ADT cannot necessarily be extrapolated to patients treated with ADT, and that rising PSA, irrespective of the absolute value, should highlight the possibility of bone metastasis in this patient population.

Although the simple nomogram that we have developed could yield useful additional information to clinicians, we recognize that our study has certain limitations. Firstly, the study population was restricted to patients who underwent conventional ^99m^Tc bone scans, which themselves have inherent limitations. These have relatively low specificity and sensitivity for detecting bone metastases compared with other imaging techniques (29), and they are rarely able to detect soft tissue or visceral metastases. Resources did not allow us to confirm that all patients lacked a positive prior bone scan. All patients with known prior positive bone scans were excluded, and as such, this tool should not be used in a patient who has a known prior positive bone scan. Secondly, this was a retrospective study based on the limited data collected non-systematically by different clinicians within a large cancer center. For example, complete data on the timing of the development of CRPC and bone metastases were not available, which could have provided useful diagnostic information. External validation of the nomogram is needed, and a prospective analysis with a clearly defined testing and treatment algorithm would yield additional valuable data to confirm and extend these findings. Thirdly, the patients in this study were referred for treatment at the Memorial Sloan-Kettering Cancer Center and may not be reflective of the types of patients managed in the community setting. In addition, the bone scan images were not obtained according to a specific schedule, and the timing of bone scan imaging may have been influenced by disease or treatment factors that were not evaluated in our chart review.

Avenues of further research became apparent during the course of this analysis. The use of ^99m^Tc bone scintigraphy as the standard first-line imaging technique is now being challenged by more sensitive and more specific modalities, such as magnetic resonance imaging and [^18^F]-fluoride positron emission tomography (PET) (30). Evaluation of other imaging techniques used to detect bone metastases in prostate cancer are already underway, most recently the use of [^18^F] sodium fluoride PET as part of the National Oncologic PET Registry. The evidence gathered from this registry will provide information on the clinical impact of sodium fluoride PET in practice and the viability of expanding reimbursement to cover sodium fluoride imaging. Therefore, nomograms that predict the risk of metastases following the use of newer as well as existing imaging techniques could also be developed. Secondly, the discriminatory power of our nomogram, as indicated by the C-index, was 0.721. Although this is comparatively high accuracy, there are clearly additional factors that contribute to the development of the M1 state. It is therefore important to identify these factors and to understand their interaction with the predictors identified in this analysis. Given that there are now several therapies developed for the treatment of M1 CRPC, including sipuleucel-T, abiraterone acetate, enzalutamide, and cabazitaxel, earlier detection may provide additional benefit for the patient. Finally, although our study did not define the transition to CRPC, patients who developed new bony metastases following the initiation of ADT have presumably already developed castration-resistant disease. Therefore, the disease variables identified as accurate predictors of a positive bone scan in this study may also be predictive of the transition from castration-sensitive disease to CRPC, which warrants further investigation.

## Conclusion

In conclusion, this study describes the first nomogram model to predict current bone scan positivity with a high level of accuracy in patients with prostate cancer who have received ADT. The nomogram could be used to highlight patients in the clinic who should be a high priority for bone scan. Therefore, integrating this nomogram into the clinical decision-making process could inform decisions on imaging and allow earlier detection of metastases, which could improve the care of ADT-treated patients.

## Author Contributions

Geoffrey T. Gotto, Changhong Yu, Melanie Bernstein, James A. Eastham, and Michael W. Kattan received no compensation related to the development of this manuscript, are fully responsible for all content and editorial decisions, and meet criteria for authorship as recommended by the International Committee of Medical Journal Editors.

## Conflict of Interest Statement

Michael W. Kattan is a consultant for GlaxoSmithKline and Merck. Geoffrey T. Gotto, Changhong Yu, Melanie Bernstein, and James A. Eastham have no financial relationships to disclose.

## Abbreviations

ADT, androgen-deprivation therapy; BCR, biochemical recurrence; CRPC, castration-resistant prostate cancer; M0, non-metastatic; M1, metastatic; PET, positron emission tomography; PSA, prostate-specific antigen; PSADT, PSA doubling time.
